# Unmasking Renal Disease in Systemic Lupus Erythematosus: Beyond Lupus Nephritis

**DOI:** 10.7759/cureus.43091

**Published:** 2023-08-07

**Authors:** Lisandra Nunez Cuello, Wendy Perdomo, Thilini Walgamage, Malsha Walgamage, Raymond Raut

**Affiliations:** 1 Department of Internal Medicine, Danbury Hospital, Danbury, USA; 2 Department of Nephrology, Danbury Hospital, Danbury, USA

**Keywords:** angiotensin ii receptor blockers (arb), angiotensin converting enzyme inhibitors (acei), systemic steroids, smoker, sle and lupus nephritis, systemic lupus erythromatosus, ig a nephropathy

## Abstract

A 64-year-old Caucasian woman with a history of hypertension and systemic lupus erythematosus (SLE) was referred to a nephrology clinic due to persistent microscopic hematuria and trace proteinuria. Initial tests showed elevated antinuclear antibodies (ANA), anti-double-stranded DNA (anti-dsDNA), and anti-Sjögren's syndrome-related antigen A (anti-SSA) levels, while other markers remained within normal limits. Over the course of a year, her urine protein-creatinine ratio increased, prompting a renal biopsy. The biopsy revealed focal crescent formation in some glomeruli and mild segmental mesangial hypercellularity in others. Although the possibility of antineutrophilic cytoplasmic antibody (ANCA)-associated nephritis with superimposed IgA nephropathy was considered, negative myeloperoxidase and proteinase 3 antibody tests led to a final diagnosis of IgA nephropathy. The patient's treatment included adding prednisone to her existing valsartan prescription for hypertension, which resulted in improved proteinuria. SLE is an autoimmune disease that can cause chronic inflammation and damage to vital organs. Approximately 50% of SLE patients may experience lupus nephritis (LN), underscoring the importance of urinalysis and renal function tests. This case presents a female patient with SLE and IgA nephropathy, a rare association that requires distinction as it affects disease management. IgA nephropathy is the most common cause of idiopathic glomerulonephritis and can lead to end-stage kidney disease in around 40% of cases. A renal biopsy is also crucial for diagnosing IgA nephropathy in patients with or without another autoimmune disease. Focal crescent formation, a histological feature observed in this case, helped exclude several diagnoses, such as lupus nephritis or pauci-immune glomerulonephritis. The primary goal of treating IgA nephropathy is to prevent disease progression. Initial treatment includes controlling blood pressure, reducing proteinuria, and implementing lifestyle modifications. Corticosteroid therapy may be considered if supportive care is insufficient.

## Introduction

Systemic lupus erythematosus (SLE) is an autoimmune disorder characterized by an augmented B and T cell response against self-antigens [1]. Approximately half of SLE patients may experience lupus nephritis, which underscores the importance of performing urinalysis and renal function tests such as serum creatinine concentration regularly. While renal abnormalities in SLE are typically attributed to lupus nephritis, it is important to consider other renal diseases that affect the general population, such as IgA nephropathy. This is a case of a patient with SLE who presented with microscopic hematuria for over one year and was diagnosed with IgA nephropathy without findings of lupus nephritis.

## Case presentation

A 64-year-old Caucasian woman has been referred to the nephrology clinic for ongoing microscopic hematuria and trace proteinuria for over one year. She has a past medical history of hypertension and SLE, diagnosed in her early twenties, which has been treated with mycophenolate mofetil, hydroxychloroquine, and prednisone. As for her social history, the patient is a smoker. Before her visit to the office, she was seen by a urologist who performed studies, including an unremarkable computerized tomography (CT) scan of the abdomen and pelvis and a normal cystoscopy. Given the concern that she could have lupus nephritis or another renal pathology, the patient underwent further investigations. 

At that time, more testing was performed, including serum anti-Sjögren's syndrome-related antigen B (anti-SS-B), anti-proteinase-3, anti-myeloperoxidase, C3 and C4 complements, renal function, and electrolyte levels. All of these were found to be within normal limits/negative. Further evaluation was conducted, involving a quantitative assessment of proteinuria and an autoimmune workup, with relevant findings outlined in Table 1.

**Table 1:** Evaluation for quantitative proteinuria and autoimmune workup. ANA: antinuclear antibodies, anti-dsDNA: anti-double-stranded DNA, and anti-SSA: anti-Sjögren's syndrome-related antigen A.

Lab investigation (units)	Results	Reference range
Urine creatinine	0.55 g/24hr	0.6-1.69
Urine protein	82 mg/24hr	<160
Total urine volume	1275 mL	600-1600
ANA	1:320	< 1:40
Anti-dsDNA	>300 IU/mL	<4
Anti-SSA	>8.0 AI	0-0.9
IgA	744 mg/dL	700-1600
IgG	3669 mg/dL	700-1600
IgM	138 mg/dL	700-1600 mg/dL

The patient was monitored for a period of one year, during which time the urine protein-creatinine ratio increased from 0.17 mg/mg Cr to 0.77 mg/mg Cr (with a normal high of less than 0.16). Initially, the patient declined a renal biopsy, but given persistent hematuria and worsening levels of urine protein-creatinine ratio, eventually the patient agreed to undergo a biopsy to obtain a histopathological diagnosis. 

**Figure 1:**
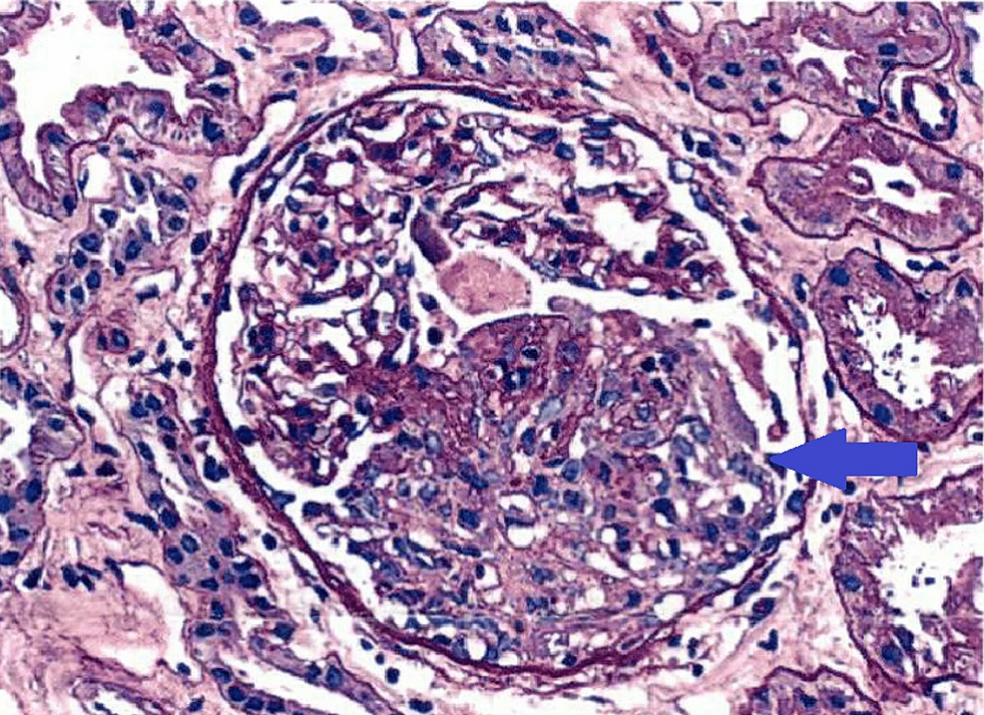
Glomeruli with cellular crescents (blue arrow) (PAS stain; 200x)

**Figure 2:**
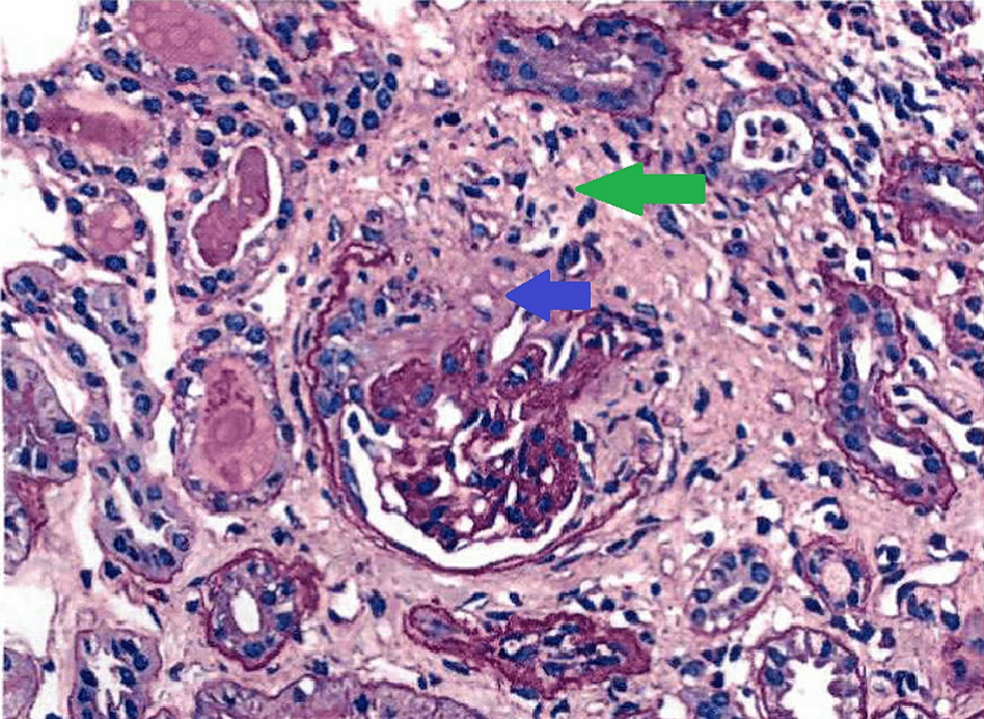
Glomeruli with fibrocellular crescents (green arrow indicates the fibrous portion, blue arrow indicates the cellular portion) (PAS stain; 200x)

The renal biopsy revealed focal crescent formation in 3 of 15 glomeruli, including cellular (Figure 1), fibrocellular (Figure 2), and fibrous. The remaining glomeruli ranged from normocellular to exhibiting mild segmental mesangial hypercellularity. Immunofluorescence reports negative IgG, IgM, C1, and C3. Based on the relatively low intensity of mesangial staining for IgA and a lack of significant endocapillary proliferation. The possibility of antineutrophilic cytoplasmic antibody (ANCA)-associated nephritis with superimposed IgA nephropathy was considered, but the patient tested negative for myeloperoxidase and proteinase 3 antibodies. Ultimately, a diagnosis of IgA nephropathy was made. Since the patient was already taking Valsartan for hypertension, it was decided to add prednisone, which led to an improvement in proteinuria.

## Discussion

SLE is an autoimmune disorder characterized by persistent inflammation and the potential for organ damage. Approximately 50% of SLE patients may experience lupus nephritis (LP), highlighting the importance of performing urinalysis and renal function tests such as serum creatinine concentration [1]. This case presents a female patient with SLE who, on biopsy, had findings consistent with IgA nephropathy. Given the rare association between SLE and hematuria due to IgA nephropathy, it is important to make the distinction between the etiology of renal disease, as it will be managed differently. Up until 2019, only eight cases of this rare association were described [2].

While renal abnormalities are typically attributed to lupus nephritis in patients with SLE, it is important to consider other renal diseases that affect the general population, including renal amyloidosis, Focal segmental glomerulosclerosis (FSGS), minimal-change disease, IgA nephropathy, infection-related glomerulonephritis (GN), and glomerulocystic kidney disease, that are not related to lupus nephritis [3].

IgA nephropathy is the most common cause of idiopathic glomerulonephritis and can progress to end-stage kidney disease in approximately 40% of cases. Although the likelihood of this condition is greater among males in their twenties and thirties, it is also essential to consider the possibility of this disease in older patients with other autoimmune conditions, as in our case [4]. In patients with IgA nephropathy and SLE, the clinical presentation is usually continuous microscopic hematuria, with or without proteinuria. In some cases, patients may develop gradual or rapid progressive kidney function impairment [1]. 

Lupus nephritis can be divided into six different subtypes, which include minimal mesangial, mesangial proliferative, focal proliferative, diffuse proliferative, membranous nephropathy, and advanced sclerosing [1,5]. Patients with active SLE and LP who undergo laboratory testing may show persistent and elevated levels of anti-dsDNA antibodies, as well as decreased levels of complement proteins C3 and C4 [6]. Our patient had elevated levels of ANA, anti-dsDNA, and anti-SSA antibodies, which were consistent with her history of SLE and active disease. Highlighting the poor guidance for diagnosing LP with only bloodwork; however, it should be used better to understand the risk of disease flare and possible renal injury. 

For an accurate diagnosis of lupus nephritis, it is advised to conduct a kidney biopsy for all patients with SLE who exhibit an acute rise in serum creatinine, proteinuria exceeding 500 mg/24 h, a urine protein/creatinine ratio surpassing 0.5 g protein/g creatinine, hematuria in conjunction with any level of proteinuria, or an active sediment/cellular cast. In a patient in whom IgA nephropathy is also suspected with or without another autoimmune disease, the definitive diagnosis will also involve performing a renal biopsy. The biopsy will allow us to assess the degree of renal involvement as well as its activity and damage [7,8]. 

Another significant finding in this case was the focal crescent formation observed on renal biopsy. Focal crescent formation is a histological feature seen in various forms of glomerulonephritis. The lack of significant endocapillary proliferation and negative immunofluorescent staining for IgG, IgM, C1, and C3 helped exclude several differential diagnoses, such as lupus nephritis or pauci-immune glomerulonephritis. The dominant deposit of IgA in the mesangium without IgM or IgG was consistent with IgA nephropathy [5].

Once the patient is diagnosed with IgA nephropathy, the primary goal is to prevent the progression of the disease. The initial treatment consists of controlling blood pressure and reducing proteinuria with renin-angiotensin system blockade. The use of sodium-glucose cotransporter 2 inhibitors for persistent proteinuria has also been described as part of supportive care [5]. Other lifestyle modifications should also be made, such as decreasing sodium and protein intake, quitting smoking, exercising regularly, and maintaining a healthy weight. If, after six months of supportive care, the patient has persistent proteinuria > 1 g/dL and a glomerular filtration rate (GFR) > 50 ml/min per 1.73 m2, then corticosteroid therapy can be considered [1,9]. 

Treatment for lupus nephritis always involves the use of immunosuppressant agents, such as corticosteroids, mycophenolate mofetil, cyclophosphamide, belimumab, and calcineurin inhibitors (such as tacrolimus and voclosporin), depending on the histopathologic classification of LP [10,11]. These treatments have been widely described for focal, diffuse, or lupus membranous nephropathy.

## Conclusions

IgA nephropathy should be considered in patients with unexplained renal pathology, particularly in those with a history of autoimmune disease. A renal biopsy is crucial in establishing a definitive diagnosis, and the primary goal of treatment is to prevent the progression of the disease. The use of lifestyle modifications, pharmacological interventions, and corticosteroid therapy should be considered in the management of IgA nephropathy, depending on the individual patient's clinical presentation and disease severity.
